# Morphological and mitochondrial changes in murine choroid plexus epithelial cells during healthy aging

**DOI:** 10.1186/s12987-023-00420-9

**Published:** 2023-03-14

**Authors:** Valentina Scarpetta, Felipe Bodaleo, Chiara Salio, Amit Agarwal, Marco Sassoè-Pognetto, Annarita Patrizi

**Affiliations:** 1 Department of Neurosciences Rita Levi Montalcini, University of Turin, 10126 Turin, Italy; 2 Chica and Heinz Schaller Research Group, Institute for Anatomy and Cell Biology, 69120 Heidelberg, Germany; 3 Department of Veterinary Sciences, University of Turin, 10095 Turin, Italy; 4 Interdisciplinary Center for Neuroscience, Heidelberg University, 69120 Heidelberg, Germany; 5 Zentrum Für Molekulare Biologie der Universität Heidelberg, DKFZ-ZMBH Alliance, Heidelberg, Germany; 6 Schaller Research Group, German Cancer Research Center (DKFZ), 69120 Heidelberg, Germany

**Keywords:** Mitochondria motility, Mitochondria speed, Tight junctions, Microvilli

## Abstract

**Background:**

Choroid plexuses (ChPs) are intraventricular structures mainly composed by specialized epithelial cells interconnected by tight junctions that establish the blood-cerebrospinal fluid (CSF) barrier. ChPs are essential to produce CSF and transport solutes from and into the brain. Deterioration of ChP function and morphology has been correlated to worsening of neurodegenerative disorders. We here map morpho-functional changes in the ChP epithelial cells during healthy aging, starting from young adult to 2-years old mice.

**Methods:**

We used a multi-tiered approach, including transmission electron microscopy (TEM), immunohistochemistry, RT-qPCR, Western Blot and 2-photon microscopy (2-PM) at multiple timepoints ranging from young adult to 2-years old mice.

**Results:**

We identified distinct morpho-functional modifications in epithelial cells of ChP starting from 8 to 12 months of age, which mostly remained stable up to 2 years. These changes include flattening of the epithelium, reduction of microvilli length and an augmentation of interrupted tight junctions. We also found a decrease in mitochondria density together with elongation of mitochondria in older mice. Morphological mitochondrial rearrangements were accompanied by increased superoxide levels, decreased membrane potential and decreased mitochondrial motility in aged mice. Interestingly, most of the age-related changes were not accompanied by modification of protein and/or gene expression levels and aged mitochondria effectively responded to acute pharmacological stressful stimuli.

**Conclusions:**

Our study suggests a long-term progression of multiple morpho-functional features of the mouse choroid plexus epithelium during adulthood followed by structural remodeling during the aging process. These findings can lead to a better understanding on how functional and morphological rearrangements of ChP are correlated during aging.

**Supplementary Information:**

The online version contains supplementary material available at 10.1186/s12987-023-00420-9.

## Introduction

The Choroid Plexus (ChP) is a highly vascularized structure located in the ventricular system of the brain, where it forms a selective interface between blood and cerebrospinal fluid (CSF). The ChP has a distinct tissue organization, which comprises: an external monolayer of specialized epithelial cells, a connective stroma and a highly permeable fenestrated capillary network. Epithelial cells are interconnected by tight junctions and form a permissive barrier, called blood-CSF fluid barrier. Different types of immune cells are also present in the ChP and play a major role in the immune surveillance of the central nervous system (CNS) [1, 2].

CSF is a “nourishing liquor” containing essential micro- and macro-nutrients, ions, and bioactive molecules that are synthesized and released by the epithelial cells of the ChP. In addition, epithelial cells transport selective peptides and proteins (e.g., leptin) from blood into CSF [3]. ChP secretome and CSF composition change throughout life, and it has been suggested that such changes interfere with the physiological regulation of brain parenchyma [4, 5]. Recently, it was demonstrated that aged brain can transfer signals via CSF that interfere with ChP production of neurotrophic molecules, giving rise to decline in cognitive function and hippocampal neurogenesis [6]. Thus, the bidirectional flow of ‘molecular information’ between ChP–CSF and the brain is essential for maintaining the brain homeostasis and is compromised in aged mice [7–9].

Early studies, in humans and rats, described age-related morphological modifications of the ChP during physiological aging, such as reduction of epithelial cell height, emergence of cytoplasmic inclusions and thickening of the epithelial basement membrane [10–12]. Surprisingly, we are only aware of one previous study that analyzed morphological alterations of mouse ChP during physiological aging. Given the importance of murine models for basic and translational research, we decided to investigate age-related morphological modifications of the ChP epithelium in C57BL/6 mice. Using transmission electron microscopy (TEM) and 2-photon microscopy (2-PM), we mapped the architectural transition of the epithelial cells starting from 2-months old (m.o.) mice until up to 2-years old mice (24 m.o.). Overall, ChP epithelium showed multiple cellular changes during adulthood and aging. Mitochondria were among the most impacted organelles, displaying noticeable shape shifting, increased superoxide levels, decreased membrane potential and decreased motility. Interestingly, most of the observed alterations were detected in 8 m.o. animals and remained stable at later stages.

## Materials and methods

### Experimental animals

Inbred C57BL/6J and C57BL/6N mice were purchased from Janvier labs and housed in both the Interdisciplinary Neurobehavioral Core (INBC) at Heidelberg University and the German Cancer Research Center (DKFZ) animal facility. Both male and female mice were included in the study. Animals were housed in static cages. No environmental enrichment toys were added to the cages, only extra Kimwipes tissue. After weaning, animals were socially housed in groups of two to five animals per cage. Animals were housed in a temperature and humidity-controlled room on a 12 h light/ 12 h dark cycle and had food and water ad libitum*.* All animal experiments were approved by the local governing regional council (*Regierungspräsidium Karlsruhe*, Germany). All methods were carried out following the German Animal Welfare Act regulations. Animal studies are reported in compliance with the ARRIVE guidelines.

### Transmission electron microscopy (TEM)

**Fig. 1:**
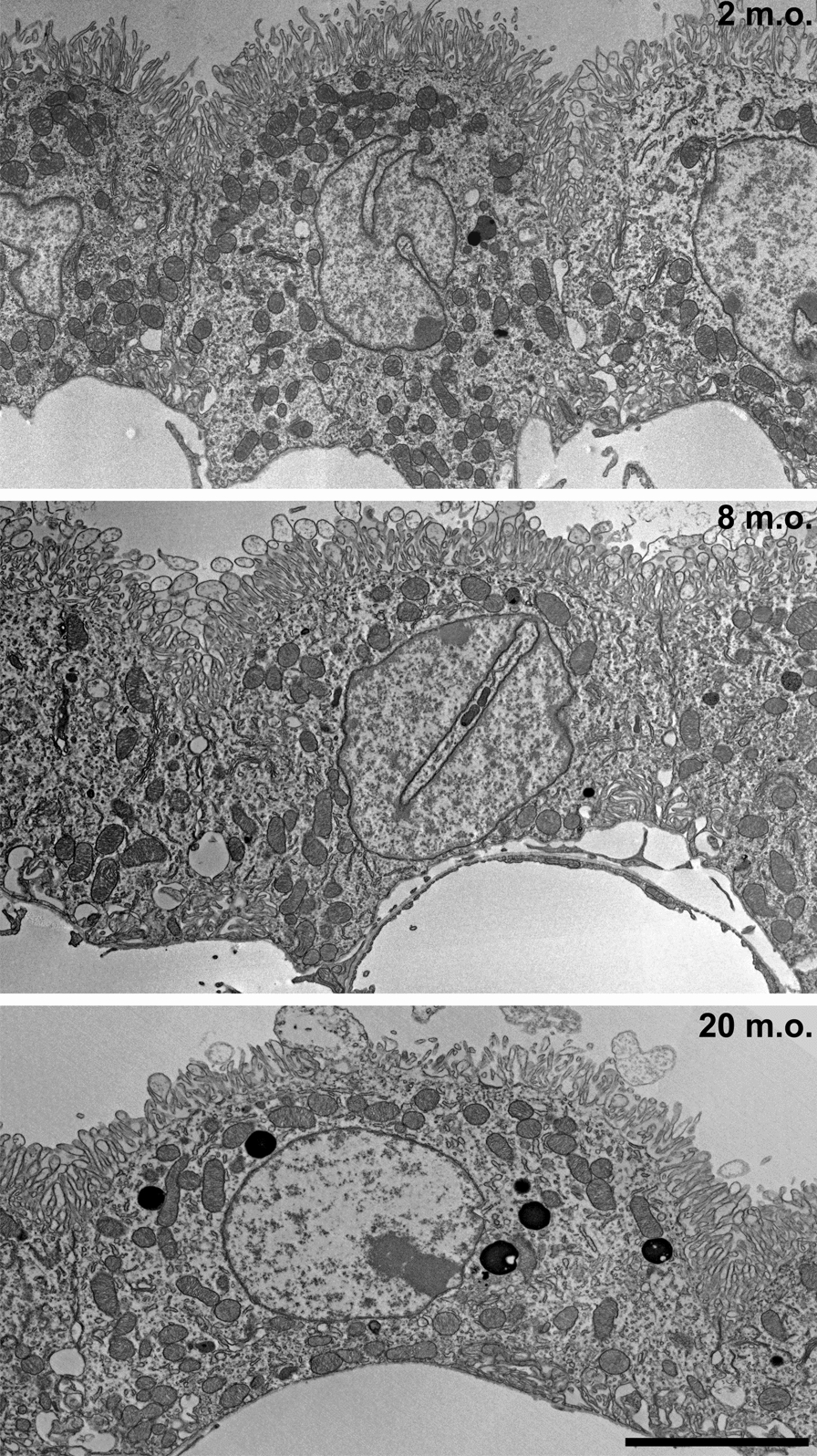
Ultrastructure of the choroid plexus epithelial cells during aging. Representative TEM images of choroid plexus epithelial cells at 2, 8 and 20 months old (m.o.). Scale bar: 5 µm

Two m.o. (n = 3), 8 m.o. (n = 3) and 20 m.o. (n = 3) animals were perfused with PBS 0.1 M (Gibco) followed by 2.5% glutaraldehyde in cacodylate buffer (CaCodylade buffer 50 mM, KCl 1 M, CaCl_2_ 0.1 M, MgCl_2_ 0.1 M, Glutaraldehyde 2.5% and sucrose 2%). The ChP was dissected and post-fixed for 1–2 h in the same fixative at 4 °C. After washing in PB 0.2 M, the ChP was post-fixed in osmium ferrocyanide (1 volume of 2% aqueous osmium tetroxide: 1 volume of 3% potassium ferrocyanide) for 1 h at 4 °C, dehydrated for 15 min in increasing concentrations of acetone (30%, 60%, 90%, 100%), incubated in acetone/Spurr resin (1:1, 30 min, 1:2, 30 min) and in Spurr resin (Sigma-Aldrich) overnight at room temperature (RT). Finally, the ChP was embedded in Spurr resin in capped 00 BEEM capsules (Electron Microscopy Sciences) for 24 h at 70 °C. Ultrathin sections were cut with an ultramicrotome (EM UC6, Leica Microsystems), collected on uncoated nickel grids (100 mesh) and counterstained for 30 s with UranyLess EM Stain and 30 s with lead citrate (Electron Microscopy Sciences). Sections were observed with a JEM-1400 Flash transmission electron microscope (JEOL, Tokyo, Japan), and images acquired with a high-sensitivity sCMOS camera. Electron micrographs in Fig. 1 were obtained with the automated montage system of the Jeol Matataki Flash camera. Shading correction and contrast adjustment of the titled images was done with Adobe Photoshop.

### TEM image analysis

Epithelial choroid plexus cells (30 epithelial cells per mouse; 90 cells per age) were selected such that both apical and basal borders of the cells were visible and nucleus was at its best fit. The analysis was performed with a semiautomatic custom macro developed by Dr. Damir Krunic (Light Microscopy Facility, DKFZ). The following parameters were considered: surface cellular area (µm^2^); cell axis ratio, obtained by dividing the horizontal and vertical cell axes; surface occupied by microvilli (µm^2^); microvilli length (µm), average of three segments traced from the apical border of the epithelial cell until the end of the microvilli (the segments were distributed at the two lateral extremes and in the center of the structure); the area (µm^2^) occupied by basolateral membrane invaginations; the percentage of invaginations distributed by size and animal age; tight junction integrity, percentage of interrupted tight junctions; total length of tight junctions (µm); tight junction contact area (%); average number of tight junction dilatations; mitochondria distribution; mitochondria localization, calculated based on their distance from the apical cell surface (the shortest distance was extracted to calculate the apical-basal proximity ratio, such that 1 corresponded to the basal surface and 0 to the apical surface); mitochondria density (mito/µm^2^); mitochondria surface area (µm^2^), average of the surface occupied by all mitochondria profiles per cell; mitochondria perimeter (µm), average of the perimeter of all mitochondria profiles per cell; circularity, calculated as 4π x Area/Perimeter^2^ (round-shaped mitochondria profile have a value close to 1, while elongated mitochondria have a score closer to 0); Feret’s diameter (µm), longest distance between any two points within the selected mitochondrion; aspect ratio, representing major to minor axis ratio of each mitochondrion.

### Tissue processing for light microscopy

Two–three m.o., 8–12 m.o. and 20–24 m.o. mice were either transcardially perfused with 4% paraformaldehyde (PFA) in sodium phosphate buffer (PBS 0.1 M, pH 7.4) or euthanized by cervical dislocation. Perfused brains were post-fixed in 4% PFA for 1 h at 4 °C, cryoprotected in 10, 20 and 30% sucrose in PBS at 4 °C and then embedded in cryostat medium (Tissue-Tek® O.C.T. Compound, Sakura). Freshly dislocated brains were rinsed with HBSS, embedded in O.C.T. and then frozen in dry ice.

### Immunostaining

All brains were sliced in 20 µm-thick coronal or sagittal sections using a cryostat (Leica CM1950). Before immunostaining, frozen sections were fixed with cold 4%-PFA for 10 min, on ice, followed by rinses with PBS 0.01 M. Sections were blocked and permeabilized (0.5% Triton X-100 and 10% normal donkey serum in PBS) for 1 h at RT, incubated in primary antibodies overnight followed by secondary antibodies for 2 h (0.5% Triton X-100 and 3% normal donkey serum in PBS). Sections were counterstained with Hoechst (D9542-1MG, Sigma Aldrich; 1:4000) and mounted using Dako Fluorescent Mounting Medium (GM304, Agilent Technologies, Inc). The following primary antibodies were used: mouse anti Ezrin (sc-58758, Santa Cruz Biotechnology; 1:500); mouse anti ATP5A (ab14748, Abcam; 1:1000); rabbit anti Claudin-1 (71-7800, Thermo Fisher Scientific, 1:1000); rat anti Zonula occludens-1 (14-9776-82, Invitrogen, 1:500); rabbit anti GLUT1 (ab116730, Abcam, 1:1000); mouse anti Na^+^/K^+^-ATPase (sc-48345, Santa Cruz Biotechnology, 1:800). Secondary antibodies were selected from Alexa series: Alexa Fluor™ 555 (A-31570, Thermo Fisher Scientific; 1:500); Alexa Fluor™ 488 (712-545-150, Jackson ImmunoResearch, 1:1000) and Alexa Fluor™ 555 (A-31572, Thermo Fisher Scientific; 1:500). For mitochondria labeling an additional antigen retrieval step with L.A.B Solution treatment (Polyscience) for 10 min at RT was applied. Images were acquired using a Zeiss LSM780 confocal microscope with × 20, × 40 or × 60 objective (oil immersion). Images were acquired with at least 1024 × 1024-pixel resolution. ZEN Black software was used for image acquisition and ImageJ (Fiji, NIH) for processing and analysis [13].

#### Confocal mean intensity analysis

For GLUT1 and Na^+^/K^+^-ATPase expression analysis, 1024 × 1024 fields of ChP were imaged using a × 20 objective. The mean pixel intensity of GLUT1 and Na^+^/K^+^-ATPase signals was measured by manually drawing regions of interest at basal and apical cellular segments, respectively, using Fiji software (*n* = 4–8 images per animal, 3 animals per age).

#### Tight junction analysis

Sholl analysis was used to quantify tight junction (TJ) organization (3 to 6 images per animal, 3 animals per age). We used a dedicated macro developed by Dr. Anthony D. Hill with the Fiji software. TJs were analyzed from images characterized by homogeneous cell distribution and staining covering at least 80% of the image surface. Signal background was removed by appropriate threshold, an initial circle was positioned in the center of the image followed by successive larger circumferences increasing of a 10 µm step. The number of intersections between the concentric circumferences and stained network was calculated.

### Immunoblotting

Two–three m.o. (n = 3), 8–12 m.o. (n = 3) and 20–24 m.o. (n = 3) mice were sacrificed by cervical dislocation. The ChP was pulled from both lateral and fourth ventricles under a dissection microscope (stereomicroscope Stemi DV4, Zeiss), frozen in liquid nitrogen and stored at – 80 °C. Tissues were homogenized in 1X RIPA Lysis reagent (9806S, Cell Signaling) supplemented with Pierce Protease and Phosphatases inhibitor mini tablets (A32955 and A32957, Life Technologies) on dry ice. Tissues were mechanically dissolved and centrifuged at 12,000 rpm for 15 min at 4 °C. Protein concentration was measured with BCA assay (23225, Life Technologies) and the supernatant was mixed with Laemmli buffer (1610747, Bio-Rad). Approximately 35 µg of proteins were loaded onto gels, electrophoresed by SDS-PAGE and then transferred on 0.45 µm nitrocellulose or PVDF membranes. Membranes were blocked for 1 h at RT with 5% non-fat dried milk dissolved in TBST buffer (Tris-buffered saline containing 0.1% Tween 20) and then incubated on a shaker overnight at 4 °C in blocking buffer containing rabbit anti GLUT1 (ab115730, Abcam, 1:1000), mouse anti Na^+^/K^+^-ATPase (sc-48345, Santa Cruz Biotechnology, 1:500), mouse anti AQP1 (sc-55466, Santa Cruz Biotechnology, 1:500), total OXPHOS antibody cocktail (ab110413, Abcam, 1:1500) that included a positive control (rat heart mitochondria). The membranes were further incubated with horseradish peroxidase (HRP)-conjugated secondary antibody for 1 h at RT. For allowing quantitative comparison and normalization, the membranes were incubated with a rabbit anti-Calnexin antibody (ab22595, Abcam; 1:10,000). The membranes were then developed using the Pierce ECL chemiluminescence substrate (32106, Life Technologies), visualized with ChemiDoc™ XRS (Bio-rad), and band density was measured by ImageJ (Fiji, NIH).

### RNA isolation

For evaluating mRNA levels, ChPs were processed as previously described [14]. For mitochondria related genes, 6 mice per age were used, while for TJs related genes 4–5 mice per age were. Briefly, mice were sacrificed by cervical dislocation and the ChP was pulled from both lateral and fourth ventricles under a dissection microscope (stereomicroscope Stemi DV4, Zeiss) and frozen in liquid nitrogen. Total RNA was extracted using RNeasy Mini Kit (Qiagen) following manufacturer’s protocol. Tissue was disrupted and homogenized using Lysis buffer RTL and QIAshredder spin columns (Qiagen). Genomic DNA was removed using RNase-free DNase I (Qiagen) for 15 min at RT. RNA was eluted with 30 µl of RNase free water. RNA quality and concentration were measured by UV spectrophotometry on NanoDrop One (Thermo Scientific). RNA samples with absorbance ratios below 1.8 were precipitated using 100% ethanol, ammonium acetate and glycogen (R0551, Thermo Scientific) following manufacturer’s protocol. RNA was reverse-transcribed into cDNA using the High-Capacity RNA-to-cDNA kit (4387406, Thermo Scientific) and cDNA samples.

### Reverse transcription quantitative real-time PCR (RT-qPCR)

RT-qPCR was performed using PowerUp SYBR Green Master Mix (A25742, Thermo Scientific) following manufacturer’s protocol, with 50 ng cDNA, 20 µM each primer in a 10 µl reaction volume. All RT-qPCR experiments were performed on LightCycler 480 (Roche) and gene expression levels were normalized to the expression of the two stable housekeeping genes *Tbp* and *Rlp13a*. The following primer sequences were used:

*Cldn1* (FW: AGCACCGGGCAGATACAGT; REV: AGCACCGGGCAGATACAGT);

*Tjp1* (FW: GCACCATGCCTAAAGCTGTC REV: ACTCAACACACCACCATTGC);

*Ndufb8* (FW: GGCCGCCAAGAAGTATAACA; REV: TGATACCACGGATCCCTCTC);

*Sdhd* (FW: TGGTCAGACCCGCTTATGTG; REV: GGTCCAGTGGAGAGATGCAG);

*Uqcrc2* (FW: AAAGTTGCCCCGAAGGTTAAA; REV GAGCATAGTTTTCCAGAGAAGCA);

*Atp5a* (FW: GGAGCCCAGCAAGATCACAAAGTT; REV: ATCTGGTGACAGTGACAGGGCTTT);

*Rlp13a* (FW: ACAGCCAAGATTCACGGTAGA REV: GCTTCTTCTTCCGATAGTGCATC)*;*

*Tbp* (FW: CCTTGTACCCTTCACCAATGAC; REV: ACAGCCAAGATTCACGGTAGA).

### Raw data processing

Non-baseline-corrected RT-qPCR raw data were extracted from the machine to provide input for LinRegPCR (version 2020.0) and analyzed accordingly to the protocol published by Hoa and Patrizi [14]. In brief, the software performed baseline correction for each sample individually and calculated amplification efficiency (E), quantification cycle (Cq) and coefficient of determination (R^2^) by fitting a linear regression model to log-linear phase. Technical replicates were examined, and arithmetic mean was taken as the Cq value representing biological samples.

### Ex vivo two-photon microscopy

ChP explants were dissected from 3–4 m.o. and 22–24 m.o. animals (*n* = 3–4 mice per age). Explants were allowed to recover in artificial cerebrospinal fluid (ACSF) containing (in mM): 119 NaCl, 2.5 KCl, 2.5 CaCl_2_, 1.3 MgCl_2_, 1 NaH_2_PO_4_, 26.2 NaHCO_3_ and Dextrose (292–298 mOsm/L), bubbled with carbogen (95% O_2_/5% CO_2_) for 10 min at 37 °C, then maintained at RT for the entire duration of the experiment. Live-cell imaging was performed using a custom built 2-PM (Bergamo II, Thorlabs, USA) with a Nikon NIR, × 60 1.0 NA objective. 2-PM excitation was achieved using a mode-locked Ti: sapphire laser (Coherent Ultra II).

#### MitoTracker loading, imaging and analysis

Explants were incubated with 100 nM MitoTracker Red CMXRos (M7512, ThermoFisher) for 30 min at RT, and rinsed 3 times with carbogen-bubbled ACSF. Ti: sapphire laser (Coherent Ultra II) was tuned at 940 nm, and each image frame was acquired at a rate of 0.52 s/frame (about 1.9 Hz) for 400 s and was 90.31 µm^2^ at 512 × 512-pixel resolution. Time series were acquired as z-stacks with a step size of 0.3 µm for 10.2 µm z-depth. For mitochondrial dynamics analysis, images were registered using ImageJ plugin *Correct 3D drift*, and mitochondrial tracking was performed manually using the ImageJ plugin *MTrackJ* [15]*.* Analyzed parameters were total distance, displacement between two consequential spatial points, and instantaneous and average speeds (*n* = 50 mitochondria per animal, 3 animals per age).

#### TMRM and MitoSOX loading, imaging and analysis

For mitochondrial membrane potential experiments, explants were either kept in ACSF or incubated with 10 µM Rotenone (HB5398, Hellobio) or with10 µM Oligomycin A (HB4488, Hellobio) for 15 min, followed by co-incubation with 25 nM TMRM (ab228569, Abcam) and 100 nM MitoTracker Green (M7514, Thermofisher) for 45 min. For mitochondrial reactive oxygen species (ROS) measurements, explants were coincubated for 45 min with 200 nM MitoSOX Red (M36008, Thermofisher) and 200 nM MitoTracker Green. For all treatments, tissues were rinsed for 10 min with bubbled ACSF before imaging. Ti: sapphire laser (Coherent Ultra II) was tuned to 860 nm, and each image frame was 111.15 µm^2^ at 1024 × 1024-pixel resolution. Z-stacks were acquired with a step size of 0.5 µm for 50.5 µm z-depth. Acquisition parameters were maintained the same within experimental groups. Fluorescence intensity was calculated by cell. Cells were randomly selected such as the nucleus was at its best fit. The analysis was manually performed. Pixel mean fluorescent intensity values were obtained using *Measure* tool in Fiji software (TMRM: 2–3 m.o., *n* = 180–250 cells, 22–24 m.o., *n* = 150–240 cells, 4 animals per age; MitoSOX: 2–3 m.o., *n* = 200 cells, 22–24 m.o., *n* = 170 cells, 3 animals per age).

### Statistical analysis

**Fig. 2:**
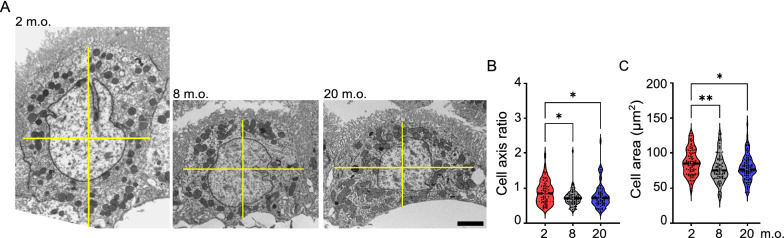
Remodeling of the gross morphology of choroid plexus epithelial cells during aging. **A** Representative TEM images of choroid plexus epithelial cells at 2, 8 and 20 months old (m.o.). Yellow lines indicate horizontal and vertical cellular axes. Quantification of cell axis ratio (**B**) and cell profile area (**C**) averaged per cell.* n* = 30 cells per animal, 3 animals per age; black dashed line: median; Kruskal–Wallis; *p < 0.05, **p < 0.01. Scale bar: 2 µm

**Fig. 3:**
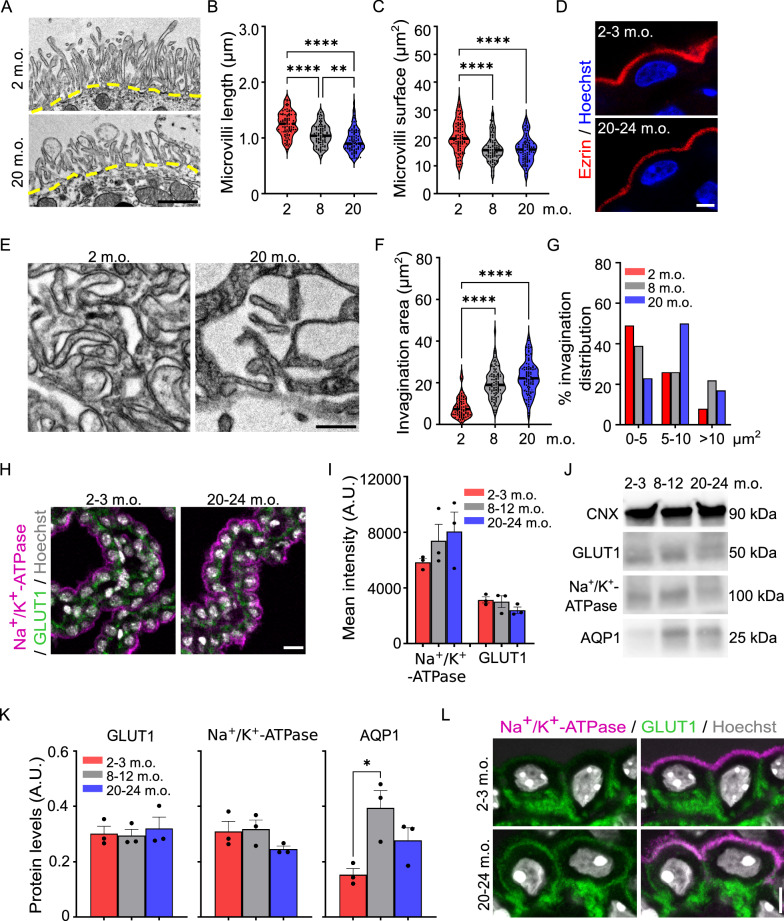
Ultrastructural and molecular remodeling of both apical and basal cellular compartments during aging. **A** High magnification TEM images showing representative microvilli at 2 and 20 months old (m.o.). Yellow dashed lines indicate the apical cell surface from which microvilli originate. Quantification of microvilli length (**B**) and surface occupied by microvilli (**C**) averaged per cell. **D** Representative images of Ezrin immunofluorescence (red) at 2–3 and 20–24 m.o. **E** High magnification TEM images showing representative basolateral invaginations at 2 and 20 m.o. **F** Quantification of the surface of basolateral invaginations. **G** Distribution in the three age groups of the surface occupied by basolateral invaginations organized by median size. **H** Representative low-magnification immunofluorescence images of Na^+^/K^+^-ATPase (magenta) and GLUT1 (green) immunoreactivities at 2–3 and 20–24 m.o. **I** Quantification of fluorescence mean intensity at 2–3, 8–12 and 20–24 m.o. A.U., arbitrary unit. **J** Western blot of GLUT1, Na^+^/K^+^-ATPase and AQP1. **K** Western blot quantification. **L** Representative high magnification immunofluorescence images of Na^+^/K^+^-ATPase (magenta) and GLUT1 (green) at 2–3 and 20–24 m.o. **B**, **C**, **F** *n* = 30 cells per animal, 3 animals per age; black dashed line: median; Kruskal–Wallis; **I**, **K** *n* = 3 animals per age; Data are mean ± s.e.m.. *p < 0.05, **p < 0.01, ****p < 0.0001. Scale bars: 1 µm (**A, E**), 6 µm (**H**), 10 µm (**L**)

**Fig. 4:**
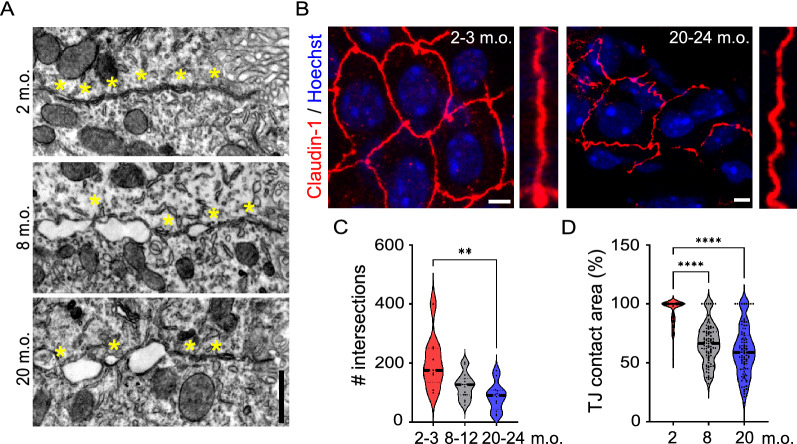
Increased frequency of interrupted tight junctions in aged choroid plexus epithelium. **A** Representative TEM images of tight junctions in 2, 8 and 20 months old (m.o.) choroid plexus epithelial cells. Yellow asterisks represent junctional contact areas. **B** Representative immunofluorescence images of Claudin-1 (red) at 2–3 and 20–24 m.o. Zoomed inserts show high-magnification Claudin-1-immunopositive tight junctions. **C** Quantification of the number of Claudin-1-immunopositive intersections at 2–3, 8–12 and 20–24 m.o. *n* = 3–6 images per animal, 3 animals per age; One-Way ANOVA. **D** Quantification of junctional contact areas (%) at 2, 8 and 20 m.o. *n* = 30 cells per animal, 3 animals per age. Black dashed line: median; Kruskal–Wallis. **p < 0.01, ****p < 0.0001. Scale bars: 1 µm (**A**), 4 µm (**B**)

**Fig. 5:**
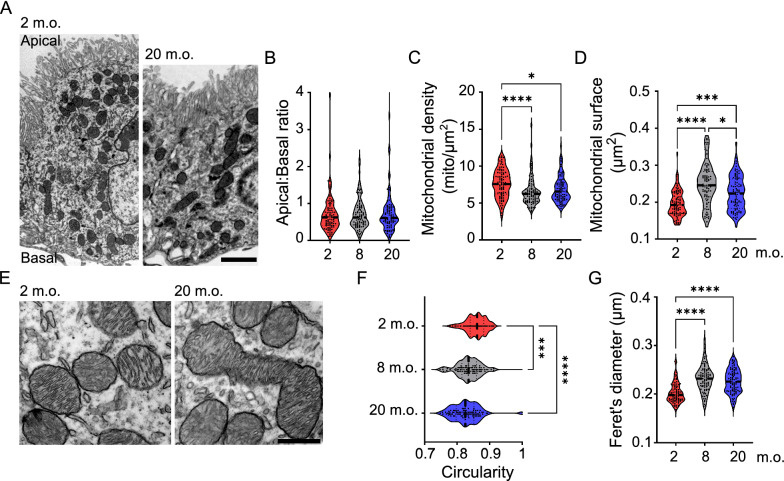
Increased mitochondrial elongation in aged choroid plexus epithelial cells. **A** Representative TEM images of choroid plexus epithelial cells at 2 and 20 months ols (m.o.). Quantification of intracellular distribution (**B**), density (**C**) and sectional surface of mitochondria (**D**). **E** High magnification TEM images showing representative mitochondria at 2 and 20 m.o. Quantification of mitochondria circularity score (**F**) and Feret’s diameter (**G**). *n* = 30 cells per animal, 3 animals per age; black dashed line: median. Kruskal–Wallis; *p < 0.05, ***p < 0.001, ****p < 0.0001. Scale bars: 2 µm (**A**), 500 nm (**E**)

**Fig. 6:**
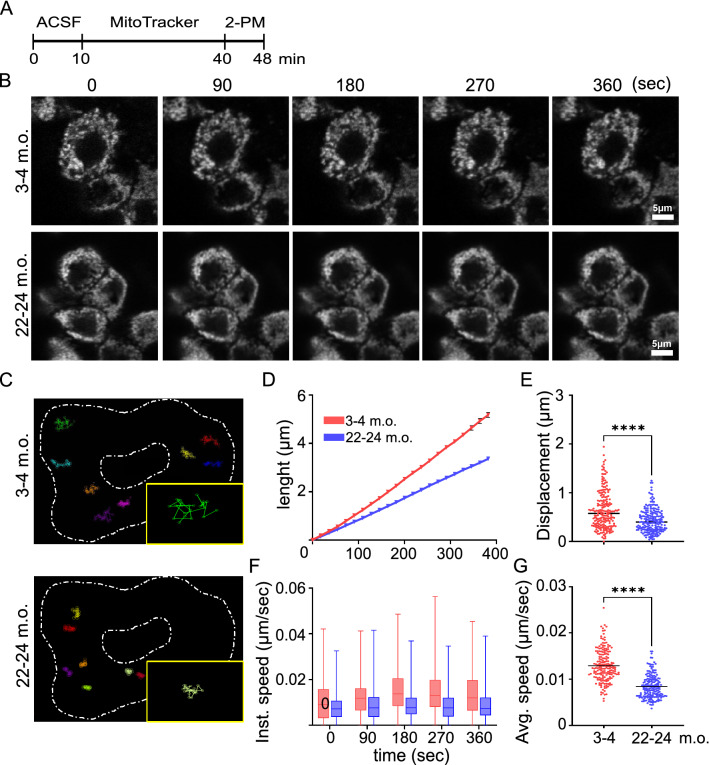
Mitochondrial movement is reduced in aged choroid plexus epithelial cells. **A** Schematic of MitoTracker incubation and 2-PM imaging (ACSF: artificial cerebrospinal fluid). **B** Representative 2-PM images of 3–4 and 22–24 months old (m.o.) epithelial cells. Scale bar = 5 µm. **C** Manually traced mitochondria in 3–4 and 22–24 m.o. cells. Inserts show a single traced mitochondrion. Quantification of total length traveled by mitochondria (**D**), mitochondrial displacement at 400 s (**E**), mitochondrial instantaneous (Inst.) speed (**F**) and mitochondrial average (Avg) speed at 400 s (**G**) in 3–4 and 22–24 m.o. *n* = 50 mitochondria per animal, 4 animals per age; black line: median. **E**, **G**: Mann–Whitney; ****p < 0.0001

**Fig. 7:**
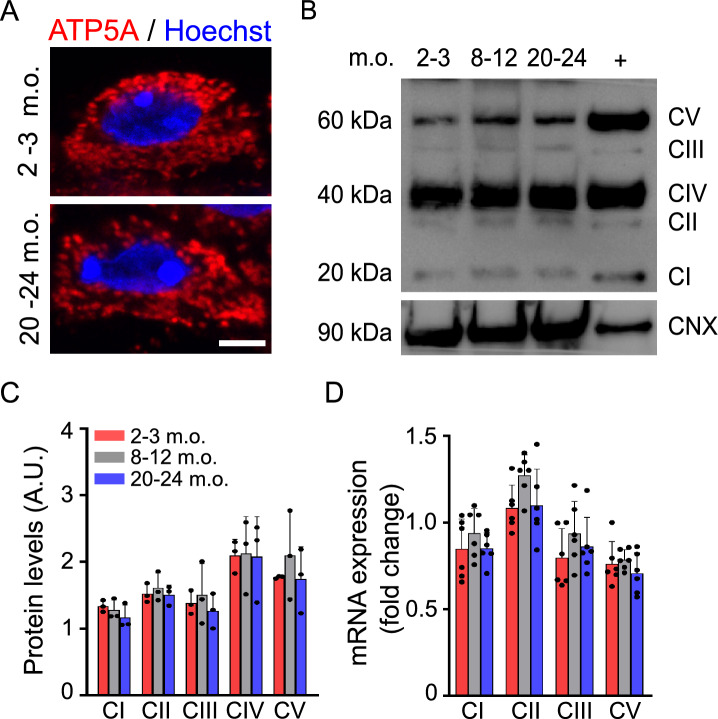
Mitochondrial biochemical composition is preserved during aging. **A** Representative immunofluorescence images of ATP5A (red) at 2–3 and 20–24 months old (m.o.). Scale bar: 4 µm. **B** Western blot of OXPHOS complex subunits from 2–3, 8–12, 20–24 m.o. choroid plexuses and positive control (+, heart). **C** OXPHOS protein expression levels at all ages. A.U., arbitrary unit. *n* = 3 animals per age. **D** Quantification of relative mRNA expression levels of OXPHOS complex subunits. *n* = 6 animals per age. Data are mean ± s.e.m

**Fig. 8:**
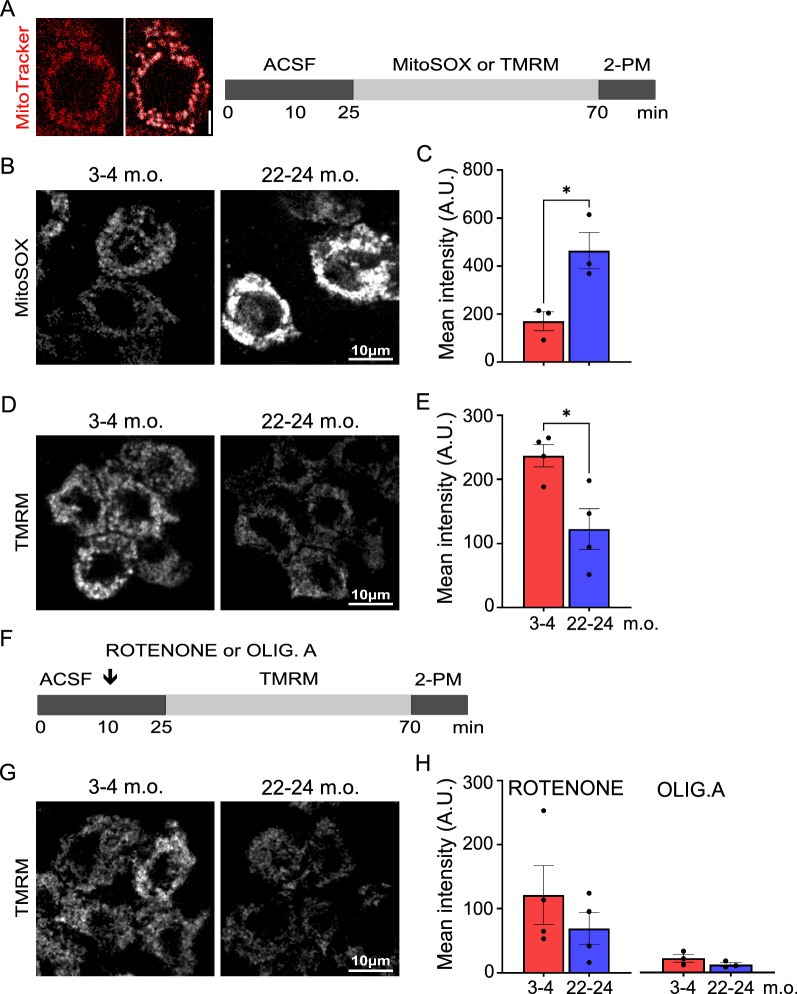
Aged choroid plexus epithelial cells display reduced mitochondrial membrane potential and increased mitochondrial ROS production. **A** Colocalization of MitoTracker Green and TMRM in choroid plexus explants (left). Schematic of MitoSOX or TMRM incubation and 2-PM imaging (ACSF: artificial cerebrospinal fluid). **B** MitoSOX representative 2-PM images of 3–4 and 22–24 months old (m.o.) choroid plexus epithelial cells. **C** Quantification of MitoSOX fluorescence mean intensity at both ages. *n* = 3 animals per age. **D** TMRM representative 2-PM images of 3–4 and 22–24 m.o. choroid plexus epithelial cells. **E** Quantification of TMRM fluorescence mean intensity at both ages. **F** Schematic of co-incubation of TMRM and rotenone or oligomycin A (OLIG. A). **G** TMRM representative 2-PM images of 3–4 and 22–24 m.o. treated choroid plexus epithelial cells. **H** Quantification of TMRM fluorescence mean intensity after treatment with rotenone (left) or OLIG. A (right). *n* = 3–4 animals per age. Data are mean ± s.e.m. Un-paired t-test, *p < 0.05. A.U., arbitrary unit. Scale bars: 5 µm (**A**), 10 µm (**B, D, G**)

Statistical analysis was performed using GraphPad Prism (version 9.4.1). Appropriate statistical tests were selected based on the distribution of data and sample size. Parametric tests (One-Way ANOVA) were used only if data were normally distributed. Otherwise, nonparametric alternatives were chosen (Mann–Whitney and Kruskal–Wallis). For the ultrastructural cellular analysis (Figs. 2, 3, 4 and 5), a nonparametric test (Kruskal–Wallis test) was chosen. For gene and protein levels evaluation (Figs. 3 and 7), One-Way ANOVA analysis was performed, followed by Tukey’s multiple comparison test. For the mitochondria motility analysis (Fig. 6), Mann–Whitney nonparametric test was selected. For the mitochondria functional analysis (Fig. 8), unpaired t-test was selected. Data are either presented as median values or as mean ± standard error of the mean (s.e.m.). p values ≤ 0.05 were considered significant (*p ≤ 0.05, **p ≤ 0.01, ***p ≤ 0.001, ****p ≤ 0.0001). The statistical test used is indicated in each figure legend.

## Results

### Remodeling of choroid plexus epithelial cells during aging

To address age-dependent morphological changes of murine ChP epithelial cells, we analyzed their ultrastructure at three time points: 2–4 m.o. (from now on referred as young adult), 8–12 m.o. (adult) and 20–24 m.o. (aged) (Fig. 1). The most obvious change observed in adult animals was a progressive flattening of epithelial cells (Figs. 1, 2A), as demonstrated by a significant decrease in cell height (Fig. 2B) and cell profile area (Fig. 2C) in the adult and aged groups. The ChP of aged mice was also characterized by an enrichment of intracytoplasmic electrodense structures (Fig. 1). These intracellular inclusions increased in density (2 m.o., 0.8 ± 0.2; 8 m.o., 1.8 ± 0.3*; 20 m.o., 2.8 ± 0.4***; Kruskal–Wallis. *n* = 50 cells, 3 animals per age) and complexity from adult to the aged stage.

The apical portion of choroid epithelial cells is covered by microvilli that project into the ventricular lumen (Figs. 1, 3A). In parallel with the remodeling of cell shape, there was a significant reduction of microvilli length (Fig. 3B) and of the area occupied by microvilli (Fig. 3C) in adult and aged mice compared with the young adult stage. When we assessed the localization of Ezrin, a structural protein component of microvilli, with confocal microscopy, we found that Ezrin remained concentrated on the apical surface of the epithelial cells [16] at both ages. However, there was a clear reduction of Ezrin-immunopositive structures in epithelial cells of aged mice compared to young adult stage (Fig. 3D), confirming the ultrastructural observations.

The basal portion of choroid epithelial cells was characterized by extensive invaginations of the basolateral membrane in aged mice (Figs. 1, 3E). The area occupied by basolateral membrane invaginations was significantly increased in adult and aged ChP compared to young adult stage. The median surface of these structures also increased with aging (Fig. 3F, G).

To start understanding whether the observed morphological alterations may correlate with dynamic and/or functional consequences, we mapped the distribution of key transporters and channels known to be selectively located at apical and basal cellular compartments. Due to the limited choice of reliable antibodies, we used glucose transporter member 1 (GLUT1) and ion pump Na^+^/K^+^-ATPase as basal and apical markers [17]. Low magnification confocal images did not reveal major differences in the distribution of these proteins in both young adult and aged ChP (Fig. 3H). Neither mean intensity analysis nor western blot quantification detected differences in the protein expression levels (Fig. 3I–K). Because it was previously reported that aquaporin 1 (AQP1) is downregulated in aged ChP [1], we measured its protein expression using western blot. Surprisingly, AQP1 was significantly upregulated in adult ChP (Fig. 3J, K). We, then, mapped the cellular localization of GLUT1 and Na^+^/K^+^-ATPase in high-magnification confocal images noticing some differences between young adult and aged epithelial cells (Fig. 3L). GLUT1 staining was more visible in the apical compartment of aged epithelial cells and Na^+^/K^+^-ATPase staining was also observable descending into the lateral compartments. These observations suggest that, despite the lack of a clear change in the molecular composition, the localization of key markers could be altered in aged epithelial cells (Fig. 3L). All together our results support the possibility that morphological cellular remodeling may be associated to molecular reorganization.

Finally, we evaluated whether aging alters the organization of tight junctions (TJs) (Figs. 1, 4A). At all analyzed ages, the junctional complex was located close to the apical side of the epithelial cells, most likely due to the presence of multiple invaginations of the basolateral membrane (Fig. 1). In young adult mice, TJs were strongly interconnected with each other, although up to 17% of the junctional profiles appeared disrupted, revealing an interrupted structure. The percentage of interrupted junctions increased considerably in epithelial cells analyzed in older animals (adult: 43%; aged: 40%) (Fig. 4A, Table 1). In parallel, we performed immunofluorescence against selective TJ components, such as Claudin-1 (Cla-1) and the TJ intracytoplasmic marker Zonula occludens-1 (ZO-1) [18]. Immunolabeling for Cla-1 and ZO-1 revealed a clear difference in the distribution of these proteins in the different mouse groups. In fact, in aged mice TJs lacked linear organization, but instead appeared irregular and pseudo-spiked (Fig. 4B). Specifically, by using Sholl analysis we quantitatively characterized the spatial organization of TJs by counting the number of intersections of the TJ network (see details in Materials and Methods). We observed a significant reduction in the total number of Cla-1-positive intersections in the adult and aged groups (Fig. 4C). Similar results were also obtained for ZO-1 (data not shown). In agreement with this, ultrastructural analysis confirmed a reduction of the total junctional contact areas starting from the adult stage (Fig. 4D). Moreover, there was a reduction of the total TJ length and an increase of the number of TJ dilatations (Table 1). To further investigate whether structural alterations could be a consequence of transcriptional changes, we tested mRNA expression levels of *Tjp1* and *Cldn1* in ChP homogenates of the three groups of mice. No age-dependent modification in these key structural components was observed (*Tjp1:* 2–3 m.o., 2.2 ± 0.9; 8–12 m.o., 1.3 ± 0.1; 20–24 m.o., 3.8 ± 1.7; *Cldn1:* 2–3 m.o., 0.9 ± 0; 8–12 m.o., 0.9 ± 0.1; 20–24 m.o., 1.2 ± 0.3; One-Way ANOVA, p > 0.05). Taken together, these data indicate the occurrence of multiple age-related changes in the cellular ultrastructure of the ChP epithelium, with prominent alterations of cell size, microvilli length and TJ organization.

**Table 1:** Measurements of gross morphological parameters

	2 m.o.	8 m.o.	20 m.o.
Cell axis ratio	0.88 ± 0.04	0.72 ± 0.03*	0.78 ± 0.04*
Cell area (µm^2^)	86.5 ± 1.99	77.6 ± 2.11**	78.84 ± 1.92*
Microvilli surface (µm^2^)	20.35 ± 0.6	16.22 ± 0.49****	15.97 ± 0.49****
Microvilli length (µm)	1.26 ± 0.02	1.05 ± 0.02****	0.95 ± 0.02****^; ##^
Invagination area (µm^2^)	8.28 ± 0.54	19.66 ± 0.76****	22.54 ± 0.84****
Inclusion density (inclusion/µm^2^)	0.64 ± 0.13	2.05 ± 0.42**	2.2 ± 0.28****
Interrupted tight junctions (%)	17	42	41
Total length tight junctions (µm)	4.6 ± 0.14	4.14 ± 0.14	3.63 ± 0.16****
Total junctional contact area (µm)	4.32 ± 0.14	2.7 ± 0.1****	2.09 ± 0.1****^; ##^
Number of tight junction dilatations	0.42 ± 0.06	1.98 ± 0.12****	2.14 ± 0.13****

Mean ± s.e.m. *n* = 30 cells per animal, 3 animals in each age group. *Statistical differences compared to 2 months old (m.o.), # to 8 m.o., Kruskal–Wallis test, *p < 0.05; **p < 0.01, ***p < 0.001, ****p < 0.0001

### Elongated and stationary mitochondria in aged choroid plexus epithelial cells

Abundant mitochondria are located in the cytoplasm of epithelial cells to sustain the intense metabolic demand of the ChP. Right after birth, mitochondrial distribution shifts from the basal to the apical compartment of the cell, likely in correlation with developmental processes of the ChP epithelium [19]. To determine whether aging also affects mitochondrial polarization we semi-automatically mapped all the mitochondria located in individual epithelial cell profiles (see Materials and Methods for details) (Fig. 5A). The subcellular distribution of mitochondria did not differ between the apical and basal compartments of epithelial cells (Fig. 5B) at the three analyzed ages, suggesting that the energy demand remains rather constant in the apical and basal domains of epithelial cells throughout animal life. On the contrary, we observed that mitochondrial density decreased significantly in adult and aged mice compared with young adult (Fig. 5C), while their surface area increased with age (Fig. 5D). Moreover, we found remarkable changes in the shape of mitochondria across all age groups (Table 2), whereas the structure of cristae remained intact (Fig. 5E). On average, mitochondria of young adult mice were round-shaped, with a circularity close to 1 (Fig. 5E, F) and an average sectional profile of 0.53 ± 0.01 µm (Table 2). Starting from the adult stage, mitochondria appeared more elongated, as determined by reduced circularity (closer to 0) (Fig. 5E, F), increased Feret’s diameter (Fig. 5G), increased aspect ratio and an average sectional profile of 0.61 ± 0.01 µm (Table 2). It is of interest to note that most of the mitochondria phenotypes appeared in adult mice and then remained stable thereafter, suggesting a possible persistent maturation of these organelles until adulthood. Taken together, these results demonstrate that age impacts mitochondrial morphology in ChP epithelial cells, with mitochondria becoming more elongated and acquiring a more complex shape in older animals.

**Table 2:** Measurement of morphometric mitochondrial parameters

	2 m.o.	8 m.o.	20 m.o.
Area (µm^2^)	0.2 ± 0.00	0.25 ± 0.01****	0.22 ± 0.00*^; #^
Perimeter (µm)	0.53 ± 0.01	0.61 ± 0.01****	0.58 ± 0.01****
Density (mito/µm^2^)	7.63 ± 0.2	6.56 ± 0.2****	6.88 ± 0.2*
Apical:Basal ratio	0.71 ± 0.05	0.72 ± 0.05	0.82 ± 0.11
Circularity	0.86 ± 0.00	0.85 ± 0.02***	0.83 ± 0.00****
Feret’s diameter (µm)	0.2 ± 0.01	0.23 ± 0.01****	0.23 ± 0.01****
Aspect ratio	1.64 ± 0.02	1.71 ± 0.02****	1.79 ± 0.02**

Mean ± s.e.m. *n* = 30 cells per animal, 3 animals in each age group. *Statistical differences compared to 2 months old (m.o.), # to 8 m.o., Kruskal–Wallis test, *p < 0.05; **p < 0.01, ***p < 0.001, ****p < 0.0001

To study how re-shaping of mitochondria could affect their dynamics, we analyzed mitochondrial motility using 2-PM in whole ChP explants. The use of the 2-PM allowed us to analyze ChP samples without inflicting damage to the specimen and reducing overall cellular stress. Whole ChP explants were incubated with MitoTracker probe, and then mitochondria were recorded for 8 min at 1.9 Hz (Fig. 6A, B). In young adult mice, motility of mitochondria was remarkably low, mostly exhibiting a Brownian-like movement (wiggling) (Additional file 1: Video S1) characterized by short-range motions as apparent from the schematic illustration of manually traced mitochondria (Fig. 6C). Interestingly, mitochondria were even less motile in older mice (Additional file 2: Video S2). Manual tracing also revealed a decrease of the total distance traveled by each mitochondrion over time between two consequent temporal points in the aged ChP compared to young adult ChP (Fig. 6B, D). Moreover, after 400 s recording, the total mitochondrial displacement from its starting point was also reduced in aged samples (Fig. 6B, E). Finally, mitochondria from aged ChP exhibited a slower dynamic compared to young adult samples, as revealed by a decrease in both the instantaneous speed at different time points (Fig. 6F) and in the average speed after 400 s recording (Fig. 6G). Overall, these results demonstrate that mitochondria are lifelong stable organelles in the ChP, displaying limited mitochondria motility in aged epithelium.

To understand whether the morphological and dynamic differences were correlated to changes in the mitochondrial molecular apparatus, we first stained against ATP5A, mitochondrial membrane ATP synthase belonging to the electron transport chain, showing no major changes in mitochondrial cellular distribution between the three animal groups. However, immunofluorescent mitochondria appeared to be larger in size in older mice, supporting electron microscopic observations (Fig. 7A). To investigate whether these morphological alterations were accompanied by changes of proteins implicated in mitochondrial oxidative phosphorylation (OXPHOS), we measured OXPHOS levels by western blot in ChP homogenates (Fig. 7B). No changes were detected in the relative protein levels of the OXPHOS complex between the three groups (Fig. 7C). To further investigate possible transcriptomic changes, we quantified mRNA expression level of key OXPHOS genes using RT-qPCR. No significant differences were observed for selected representative genes for complex I (CI, NDUFB8), II (SDHD), III (UQCRC2) and V (ATP5A) (Fig. 7D), suggesting that neither transcriptomic nor protein composition is affected during aging.

Finally, we investigated key mitochondrial functional properties, such as membrane potential and levels of ROS, combining the use of gold standard probes and 2-PM in whole ChP explants. MitoTracker Green was used to determine the localization of mitochondria in epithelial cells (see material and methods for details) (Fig. 8A). First, we measured the mitochondrial superoxide levels by MitoSOX application. As expected, aged ChP showed a significant increased level of mitochondrial ROS when compared to young adult explants (Fig. 8A–C). Secondly, we measured the ability of active mitochondria to accumulate TMRM at both ages. Aged ChP showed a decreased mitochondrial membrane potential as revealed by a significantly lower TMRM accumulation compared to young adult explants (Fig. 8A, D, E), suggesting a lower capability of mitochondrial ATP synthesis. The application of rotenone and oligomycin A, selective inhibitors of complex I and ATP synthase activity, reduced the accumulation of TMRM at both ages with a similar kinetic (Fig. 8F–H), indicating that young adult and aged ChP mitochondria can respond to the same acute stressful event analogously. All together, our data demonstrate that aged mitochondria are stable organelles that, despite showing some hallmarks of aging such as high ROS levels, can effectively buffer acute stressful events.

## Discussion

In this study, we have investigated structural changes occurring in the ChP during the healthy aging process, from young adult mice to 2-years old mice. One of the major findings is that the majority of identified morphological changes occur already at the adult stage (8–12 months) and remain rather stable thereafter. Interestingly, previous studies on human samples have demonstrated that many of the morphological aged-related changes become apparent in samples from middle-aged individuals (45 years old) [20]. In addition, a recent human study confirmed that ChP transcriptomic differences become apparent at 50 years and stay stable until 80 years [21]. Together, these observations indicate that both in humans and in mice morpho-functional changes in the ChP occur relatively early at the adult stage, preceding the period more commonly associated with senescent brain [22]. In the future, it will be important to understand why age-related changes in the ChP starts early during lifetime and how this event impacts overall ChP function.

As previously reported for human and rat, we showed that mouse ChP epithelial cells exhibit a progressive cellular flattening. Atrophy is one of the most common aging phenotypes of epithelial organs, consisting in a shrink of epithelial cells and an increase in connective tissue [12, 20, 21, 23–27]. In the ChP, such structural changes have been correlated with functional variations, such as decreased enzymatic activity, both during healthy aging [28] and in degenerative disorders of the nervous system [7, 29, 30]. Dynamics studies of the CSF revealed diminished CSF production and turnover in aged brains [31, 32], decreased protein concentration starting from young adult age, and changes of the ChP secretome over time [33, 34]. We also demonstrated a significant reduction of microvilli length, essential to support flux of solutes and water from plasma to CSF [3], suggesting a decrease of the contact interface between the CSF and the epithelium [12]. On the contrary, we found that the basolateral membrane invaginations increased in size and covered a bigger cellular section in aged ChP. These structures are known to play a crucial role in blood filtration in the kidney [35], it is therefore suitable to assume that they may play a similar role in the ChP. It was previously shown that immunofluorescence staining of some choroidal proteins and transporters involved in CSF production (e.g. carbonic anhydrase II, AQP1 and Na^+^-K^+^-ATPase) is reduced in aged rats [16]. Interestingly, our analysis did not detect changes in the protein expression level of either apical or basal markers of aged epithelial cells, but it distinctly showed a possible rearrangement of their cellular distribution. In conclusion, we demonstrated that two cellular compartments dedicated to CSF production are structurally remodeled in different directions throughout the mouse life. In the future, it will be important to understand the relationship between CSF milieu and ChP cellular remodeling and how this relationship is maintained in different species.

The epithelium flattening was also accompanied by the appearance of cytoplasmic electron-dense structures, which progressively increased in density and size starting from the adult stage. These inclusions are presumably of lipid nature, like shown in studies on human samples [11]. Healthy human samples and Alzheimer’s disease patients are also characterized by the presence of Biondi ring tangles. Interestingly, the onset of these type of accumulation starts around 45 years of age in healthy samples and peaks at 70 years of age in Alzheimer’s disease patients [11, 36]. Despite the early age onset in human samples, we did not observe Biondi ring tangles in mice ChP.

The blood-CSF barrier ensures a well-controlled environment of the brain, which is necessary for proper functioning of the CNS. TJs that are present between the ChP epithelial cells control paracellular transport between blood and CSF [37, 38]. Therefore, dysregulation of TJs could result in CSF compositional changes. Interestingly, we found discontinuous TJ strands and paracellular dilatations in adult and aged ChP compared to the young adult stage. In addition, the ultrastructural analysis showed that the surface of the contact area between adjacent cells was also reduced with aging. Interestingly, the aged-related morphological phenotypes were not accompanied by changes in neither protein nor mRNA expression levels of major TJ molecular components. In agreement, a study in sheep showed a small increase in protein permeability for the blood-CSF barrier but not a complete disruption of the barrier in aged animals [39, 40]. In humans, starting from the age of 45 years old, an increased variability in the CSF/serum albumin ratio has been observed [41–43]. To our knowledge, our study is the first one demonstrating morphological reorganization of TJs in the ChP during aging. We, here, suggest that minor but critical TJ morphological rearrangements can be responsible for functional barrier permeability, as already been shown for the blood–brain barrier and also for other organs, such as the intestine [44, 45]. In the future it will be important to understand which changes are responsible for triggering such events and how the morphological TJ rearrangements influence the CSF composition.

Because of their secretory function, ChP epithelial cells contain a substantial number of mitochondria, that are essential to maintain the cellular high demand of energy [2]. Here we showed that ChP mitochondria in adult epithelial cells are characterized by a small but significant decrease in total density and by an increase in size. Similar age-related changes in mitochondria structure have been described in other organs (muscle, liver, heart and kidney) [46]. Notably, ChP mitochondria shifted from round-shaped to elongate-shaped in adult mice compared to young adult animals. These differences were maintained stable in aged ChP. In vitro models, elongated mitochondria most commonly display improved mitochondrial function [47, 48], which has been interpreted as an adaptive mechanism to sustain cell survival [48]. The structural re-organization of the mitochondria was accompanied by a significant increase in the level of mitochondrial superoxide, suggesting possible mitochondria oxidative damage, a hallmark of aging [49]. The significant reduction of TMRM accumulation in aged ChP mitochondria indicates a disruption of the mitochondrial membrane potential [50], indirectly suggesting that the ability to synthetize ATP through oxidative phosphorylation may be impaired at baseline. Interestingly, when a pharmacological acute stressful stimulus was introduced, young adult and aged ChP explants buffered it in a similar manner. It is, therefore, tempting to speculate that the morphological remodeling observed in mitochondria of the ChP may represent an adaptive mechanism engaged by ChP epithelial cells to endure stressful events.

The implementation of intact ChP explants and 2-PM microscopy allowed us to demonstrate, for the first time, that ChP mitochondria show slow and short-range movements, generally defined as wiggling movements [51], that significantly decrease with age. A similar trend has already been described in other cell types [52, 53], e.g. vascular smooth muscle cells and cardiac myoblasts, in which the reduced mobility has been partially correlated with an increase of mitochondrial size [53]. Interestingly, both mitochondrial motility and size can be regulated by cytoplasmic Ca^2+^ and can rearrange the spatial pattern of ATP production influencing the cell functionality [54]. Interestingly, ChP epithelial cells do exhibit spontaneous Ca^2+^ transients [55] and our ongoing studies indicate that the Ca^2+^ transients can change throughout mouse lifetime (data not shown), raising the possibility that intracellular Ca^2+^ dynamics could influence mitochondrial shape and motility in aged ChP epithelial cells.

In conclusion, this study provides an extensive description of morphological changes of ChP epithelial cells during aging, highlighting that most of them occur as early as 8 months and stay stable thereafter. We found that aging phenotypes of ChP epithelial cells comprise not only cellular flattening but also an enlargement of basolateral invaginations and the emergence of interrupted TJs. In addition, we demonstrated, for the first time, that the morphology of mitochondria changes during adulthood, becoming stable at 8 m.o. and stationary in aged ChP epithelium. Finally, we revealed that aged ChP exhibit a lower mitochondrial membrane potential compared to younger ages, although mitochondria responses to an acute challenging stimulus did not differ. In the future it will be important to understand how these morpho-functional phenotypes can adjust to chronic stressful events and other functional reorganizations.

## Supplementary Information

**Additional file 1: Video S1.** Mitochondria motility in young adult ChP explant.

**Additional file 2: Video S2.** Mitochondria motility in aged ChP explant.

## Data Availability

All data generated during this study are included in this published article. The developed analysis tools are available from the corresponding author upon request.

## Abbreviations

ACSF: Artificial cerebrospinal fluid
ATP: Adenosine triphosphate
ChP: Choroid plexus
Cla-1: Claudin-1
CNS: Central nervous system
CSF: Cerebrospinal fluid
OXPHOS: Mitochondria oxidative phosphorylation
ROS: Reactive oxygen species
TEM: Transmission electron microscopy
TJ: Tight junction
ZO-1: Zonula occludens 1
2-PM: 2-Photon microscopy
